# Mice with a Conditional Deletion of the Neurotrophin Receptor TrkB Are Dwarfed, and Are Similar to Mice with a MAPK14 Deletion

**DOI:** 10.1371/journal.pone.0066206

**Published:** 2013-06-11

**Authors:** Michele R. Hutchison

**Affiliations:** Department of Pediatrics, University of Texas Southwestern Medical Center, Dallas, Texas, United States of America; University of Western Ontario, Canada

## Abstract

Long bone growth results from ordered chondrocyte development within the cartilagenous growth plate. Chondrocytes are recruited from a resting pool to proliferate along the long axis of the bone, until various signals trigger differentiation and hypertrophy. We have shown previously that the neurotrophin receptor TrkB is expressed in growth plate chondrocytes, where the tyrosine kinase receptor regulates the pace of hypertrophic differentiation by modulating the activities of ERK and p38 MAP kinases. To investigate the physiological relevance of TrkB to bone growth *in vivo*, we generated mice with a targeted disruption of the receptor, and compared them to mice targeted for MAPK14, the gene for p38α. The TrkB mutant and p38α mutant mice showed a similar degree of dwarfism and delayed hypertrophic differentiation. To extend these findings, we showed that both the TrkB and p38α mutant mice have altered expression of Runx2 and Sox9, two key transcription factors required for skeletogenesis. The data provides *in vivo* evidence for the role of TrkB in bone growth, supports the role of p38 downstream of TrkB, and suggests that Runx2 and Sox9 expression is regulated by this pathway at the growth plate.

## Introduction

The rate of longitudinal bone growth is determined by the pace of endochondral ossification that occurs within the cartilaginous growth plates located at the ends of long bones and vertebrae [1]–[3]. Resting chondrocytes within the reserve zone are recruited to enter the proliferative zone, wherein they divide along the long axis of the bone. A large number of factors, acting mainly in a paracrine manner, signal the cells to cease proliferation and begin differentiating within the pre-hypertrophic zone; terminally differentiated cells are found in the hypertrophic zone, wherein glycogen accumulation leads to dramatic cell hypertrophy. We previously reported that the TrkB receptor tyrosine kinase and its ligand, brain-derived neurotrophic factor (BDNF) are expressed in growth plate chondrocytes, where their interaction inhibits proliferation and promotes chondrocytic differentiation [4].

TrkB is widely expressed in neuronal tissue, where BDNF regulates neuronal survival and differentiation in peripheral and central nervous systems, and maintains synaptic plasticity, particularly in the hippocampus and hypothalamus [5]–[7]. TrkB and BDNF are also expressed in non-neuronal cells such as vascular endothelial cells, immune cells, and osteoblasts [8]–[10]. When activated, TrkB stimulates MAP (mitogen-activated protein) kinase pathways, which occupy a critical place in many intracellular pathways that transfer extracellular signals to intracellular effectors such as transcription factors [11], [12]. Whereas in neural tissues TrkB activates the ERK MAPK pathway, in growth plate (GP) chondrocytes the ability of TrkB to enhance hypertrophic differentiation requires the increased activity of p38 MAPK and reduced activity of ERK. In both primary bovine GP chondrocytes and the cell line ATDC5, BDNF attenuates ERK activity while increasing that of p38, and BDNF-induced chondrocytic differentiation is blocked by p38 inhibition. The *in vivo* significance of these observations has not been demonstrated previously. In the present study we generated mice with targeted disruptions of either TrkB or MAPK14 (the gene for p38α), and demonstrated that the mice are similarly dwarfed. The dwarfism is due to impaired transition to hypertrophy, as cell proliferation within the growth plate was unaffected. The TrkB mutant mice have reduced expression of p38α and reduced p38 activation at the GP. Thus TrkB, acting through p38α, is required for normal long bone growth in mice.

## Results

### Dwarfism in *TrkB^flox/flox^;Col2a1-cre* mice

**Figure 1:**
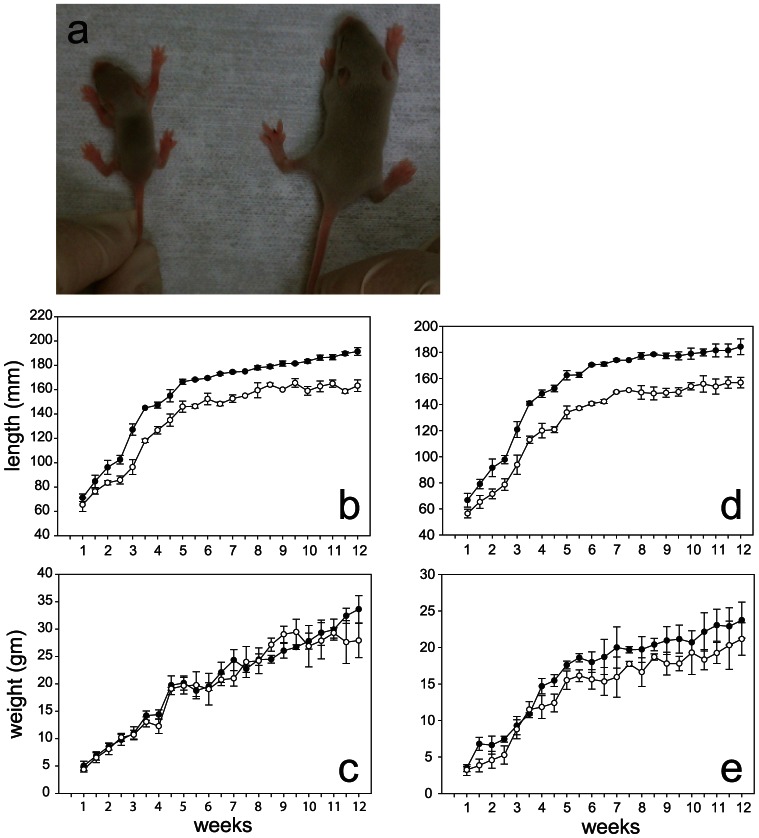
Growth defects in *TrkB ^loxp/loxp^;Col2a1-cre* mice. A, Gross appearance of TrkB mutant female and control female littermate at 10 days of age. B–D, mean nose-to-tail lengths and body weights ±SD for male (B,C) and female (D,E) mice from 1 to 12 weeks after birth; *TrkB ^loxp/loxp^* (•), *TrkB ^loxp/loxp^;Col2a1-cre* (○), n = 23 for mutants, 22 for controls.

Because *TrkB−/−* mice die within 3–4 days of life [5], [6], the cre recombinase approach was used to conditionally inactivate TrkB in cells of chondrocytic lineage by crossing *TrkB^flox/flox^* mice with *Col2a1-cre* transgenic mice. At every generation, *TrkB^flox/flox^* mice were crossed with *TrkB^flox/flox^;Col2a1-cre* mice containing only a single Col2a1-cre transgene. The *TrkB^flox/flox^;Col2a1-cre* mice (hereafter designated as mutants) were obtained at the expected Mendelian ratio of 50%. The Col2a1-cre transgene is thought to be active as early as 8.5 dpc [13]. All mutant mice were initially viable; however, approximately 20% of the females were severely dwarfed at birth (**Fig. 1a**), and none of these extremely dwarfed females survived past 2 weeks. Most of these severely dwarfed mice did not survive past post-natal day (P) 3. Similar severe dwarfism at birth was not noted among the male mutants, who demonstrated growth defects at age 3.5 weeks. The remaining female mutants showed abnormal growth by 4.5–5 weeks of age. By 12 weeks of age, nose-to-rump lengths and nose-to-tail lengths of mutant males were approximately 70–75% that of the *TrkB^flox/flox^* males; however, there was no difference in body weights. Female mutants by 12 weeks were approximately 80% the size of the *TrkB^flox/flox^* females.

### Dwarfism in *MAPK14^flox/flox^;Col2a1-cre* mice

**Figure 2:**
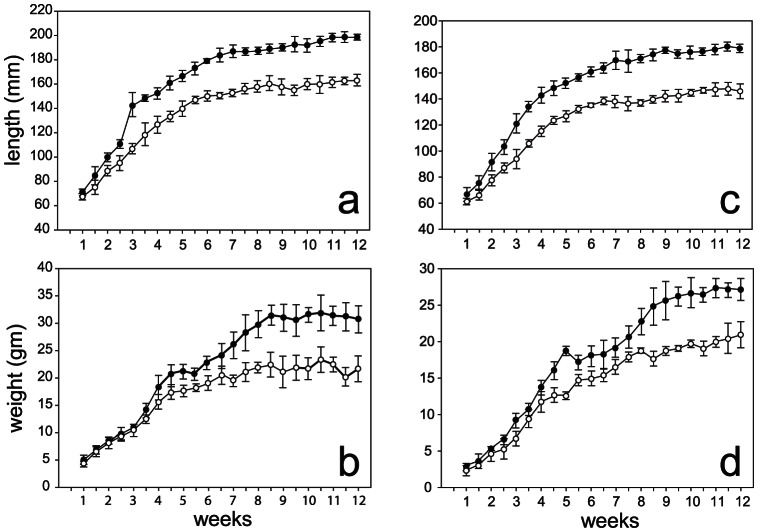
Growth defects in *MAPK14 ^loxp/loxp^;Col2a1-cre* mice. Mean nose-to-tail lengths and body weights±SD for male (A,B) and female (C,D) mice from 1 to 12 weeks after birth, *MAPK14 ^loxp/loxp^* (•), *MAPK14 ^loxp/loxp^;Col2a1-cre* (○), n = 19 for mutants, 20 for controls.

Whereas global deletions of p38 isoforms beta, gamma and delta lead to fertile mice with no discernible phenotype [14], [15], global deletions of the alpha isoform (MAPK14) are early embryonic lethal [16]. We crossed the floxed MAPK14 mice with the *Col2a1-cre* mice to conditionally delete p38α from the growth plate. The *MAPK14^flox/flox^;Col2a1-cre* mice were also obtained at the expected Mendelian ratio of 50%. Dwarfism in both genders was apparent by 3–4 weeks of age, and by 12 weeks of age weights, nose-to-rump lengths and nose-to-tail lengths of mutant males were 75–80% that of the *MAPK14^flox/flox^* mice (**Fig. 2**). The severe dwarfism seen in some *TrkB^flox/flox^;Col2a1-cre* females was not noted in the p38α mutant mice.

**Figure 3:**
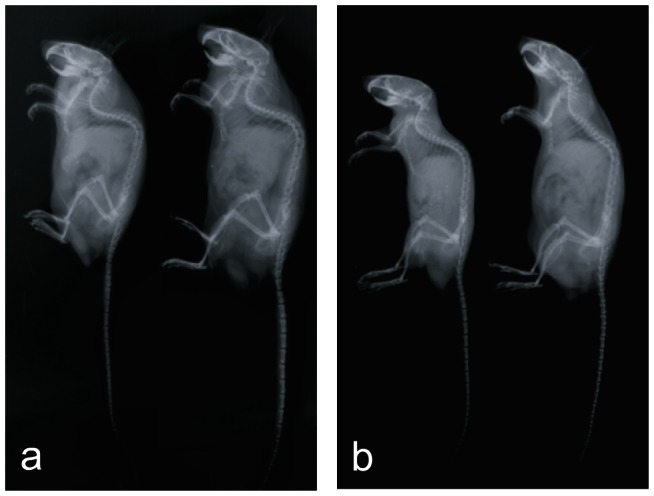
Radiographic analysis of 6 month old male TrkB mutant and control littermate (A), and p38α mutant and control littermate (B). Exaggerated kyphosis is post-mortem.

Soft X-ray analysis showed that all long bones and vertebrae were equally affected in the TrkB mutant mice (**Fig. 3a**). Lengths of tibiae, femurs and vertebrae in mutant males at 6 months of age were approximately 75–78% that of their normal littermates. The naso-occipital length of the calvarium was not significantly affected. As was seen for the TrkB mutant mice, radiographic analysis of the p38α mutants showed femur length to be approximately 78% of normal, with tibial and vertebral lengths about 75% that of their normal littermates (**Fig. 3b**). Whereas the p38α mutant mice had body weights concordant with their lengths, the TrkB mutant mice were the same weight as the control mice, making them proportionally heavier; this difference in body weights is seen in the radiographs in Figure 3.

### Growth plate abnormalities in *TrkB^flox/flox^;Col2a1-cre* mutant mice

**Figure 4:**
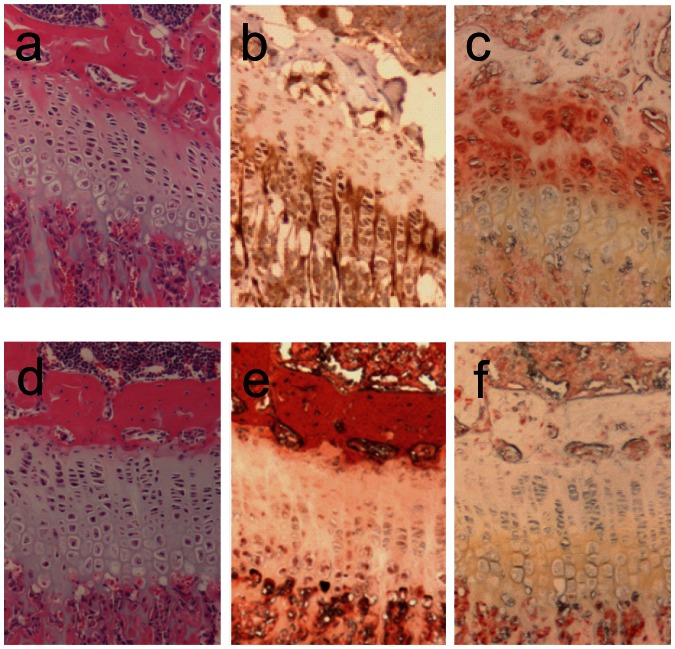
Histological analysis of long bones of TrkB mutants. A, B, and C are stained histological sections from a 4 week old male *TrkB^loxp/loxp^* control; D, E and F are from a male *TrkB^loxp/loxp^;Col2a1-cre* littermate. A, D, H&E staining; B, E, ColX immunostaining; C, F, TrkB immunostaining.

Tibiae and femurs of mice at 4 weeks of age were examined histologically. The overall morphology of the GPs from the mutant mice was similar to that of the *TrkB^flox/flox^* mice, but significant differences were apparent. The width of the GP at each age examined of male mutant mice was 78.8±5.7% (*p*<0.01) of *TrkB^flox/flox^* males. When the percentage of the GP made up of hypertrophic cells was assessed, the male mutant mice consistently showed a reduction in the width of the hypertrophic zone (HZ), such that in *TrkB^flox/flox^* mice the HZ was 51.8±4.2% of the total width, whereas the mutant mice displayed a HZ width that was 40.2±3.7% of the epiphyseal width (*p*<0.02 for both comparisons). Thus the total width of the reserve, proliferative and pre-hypertrophic zones was unchanged in the mutant mice. Immunohistochemical staining for TrkB was present in the reserve and proliferative zones of the *TrkB^flox/flox^* mice, but reduced in the mutant mice (**Fig. 4**). Staining for BrdU to assess for cell proliferation showed that, within the proliferative zone, the percentage of positive cells was not significantly different between the *TrkB^flox/flox^;Col2a1-cre* mutant mice and their *TrkB^flox/flox^* littermates (not shown).

### Growth plate abnormalities in *MAPK14^flox/flox^;Col2a1-cre* mutant mice

**Figure 5:**
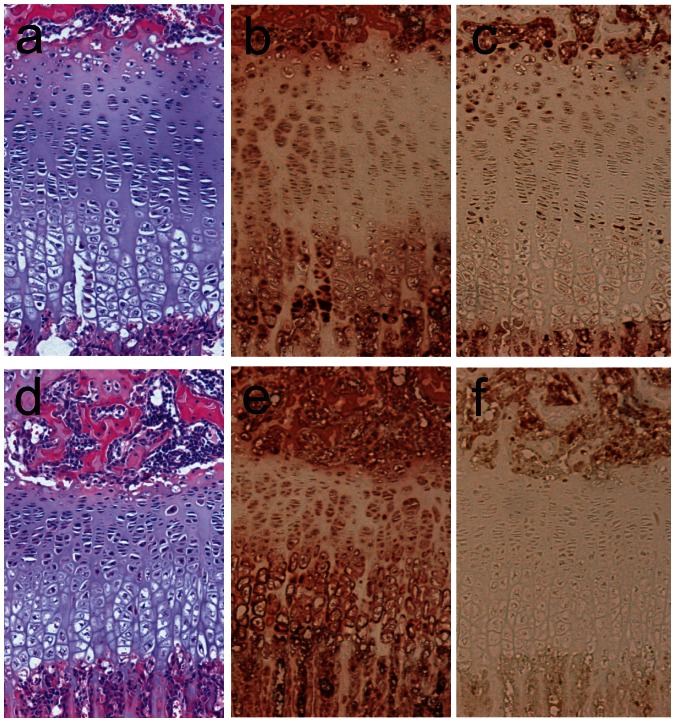
Histological analysis of long bones of p38α mutants. A, B, and C are stained histological sections from a 4 week old male *MAPK14^loxp/loxp^* control; D, E and F are from a male *MAPK14^loxp/loxp^;Col2a1-cre* littermate. A, D, H&E staining; B, E, ColX immunostaining; C, F, p38 immunostaining.

Tibiae at 4 weeks were examined; as seen in the TrkB mutant mice, the GP morphology of the MAPK14 mutant mice was normal, but staining for p38, normally localized to the proliferative and pre-hypertrophic zones, was largely absent in the mutant mice (**Fig. 5**). Overall width of the GP in mutant mice was reduced to 75.4±4.9% (*p*<0.01) that of normal littermate controls. Unlike what was seen in the TrkB mutant mice, the MAPK14 mutant mice showed narrowing of both the proliferative and hypertrophic zones. The total number of BrdU positive cells appeared to be reduced in the p38α mutant mice, but the percent of positively staining cells within the proliferative zone was not significantly different from control mice (not shown).

### Inhibition of TrkB signaling reduces expression of Sox9 and Runx2 in vitro

**Figure 6:**
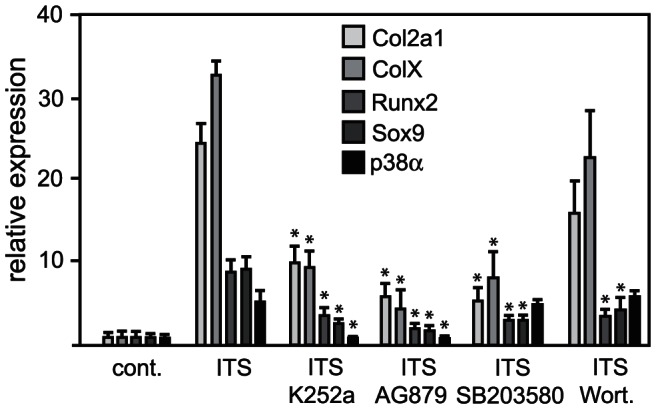
Effect of TrkB inhibition on Runx2 and Sox9 expression in ATDC5 cells. The murine chondrocytic osteosarcoma cells were cultured in differentiation media containing 10 µg/ml insulin and 25 µg/ml ascorbic acid; media was changed every other day for 6 days, at which time the indicated kinase inhibitors were added for an additional 3 days. Real-time RT-PCR was used to quantify mRNA levels of the marker proteins. Data points were calculated using the ΔΔCt method and represent the mean ±SD of real-time data from five sample pairs, expressed as fold difference from insulin alone (the calibrator). *, *P*<0.001.

To determine whether TrkB signaling is necessary for the expression of Sox9 and Runx2 –transcription factors known to be essential for growth plate development– we initially used the ATDC5 cell line, which in the presence of high-dose insulin and 25 µg/ml ascorbate recapitulates the entire chondrogenic program over a 14 day period [17], [18]. Because we were interested in the zone at which the transition from proliferation to differentiation occurs, we focused on the analogous time frame in differentiating ATDC5 cells, which in our hands is between 6–9 days after insulin addition. Cells were cultured in the presence of insulin for 6 days prior to the addition of two TrkB inhibitors, K-252a and AG879. We have previously shown that activation of the MAPK p38 is necessary for TrkB function in chondrocytes, so the p38 inhibitor SB203580 was included for comparison. Both of the TrkB inhibitors showed changes in levels of expression of chondrocyte-specific markers that were similar to that seen with the p38 inhibitor. ColX and Col2a1 RNA levels were markedly reduced after incubation from day 6–9 with either the TrkB or p38 inhibitors, as compared to levels seen after 9 days in the presence of insulin with no inhibitors (**Fig. 6**). The Trk inhibitors reduced Sox9 and Runx2 RNA levels to a similar degree as seen with the p38 inhibitor SB203580. Wortmannin was included as a general kinase inhibitor control because we had previously seen no affect of this PI3K inhibitor on ColX or Col2a1 mRNA levels [4]. Again, Wortmannin did not affect ColX or Col2a1 RNA levels, but transcript levels for Runx2 and Sox9 were decreased, suggesting that PI3K might have a role in regulating the expression of these transcription factors.

### Disruption of TrkB or MAPK14 reduces expression of Sox9 and Runx2 in vivo

**Figure 7:**
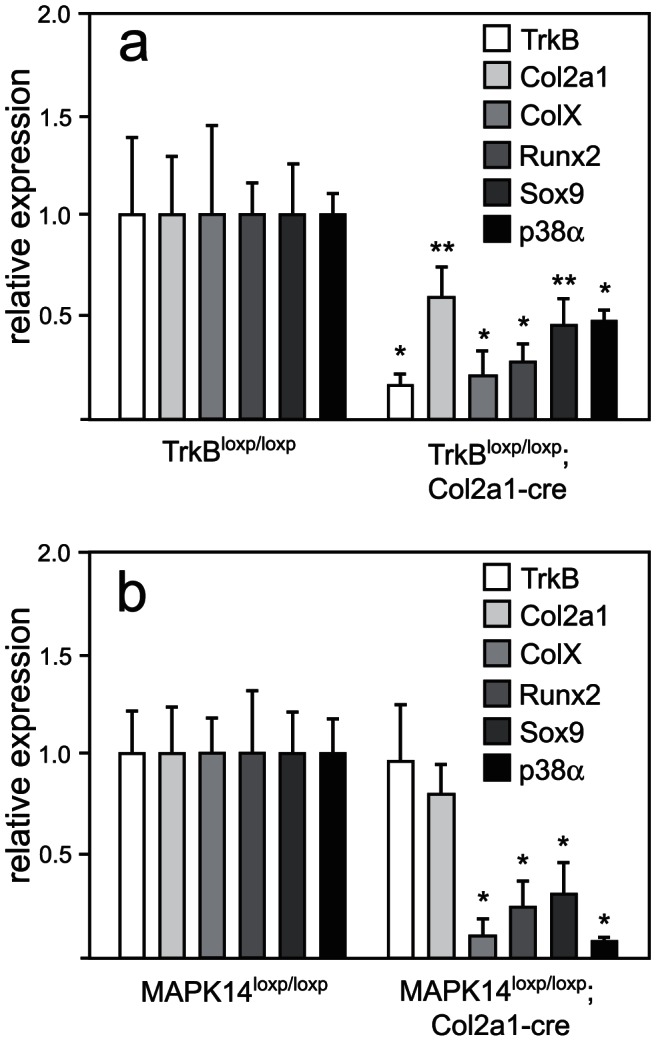
Expression of Runx2 and Sox9 in the growth plates of mutant mice. The growth plates from the proximal tibias of mutant and control littermates at day 7 after birth were dissected under a microscope, and isolated RNA analyzed by real time RT PCR for the expression of the two transcription factors as well as Col2a1, ColX, TrkB, and p38α. A, TrkB mutants compared to control littermates; B, p38α mutants compared to control littermates. Data points represent the mean ±SD of six samples, expressed as fold difference from control samples (calibrator); *, *P*<0.001, **, *P*<0.01.

To determine whether disruption of TrkB would also affect the expression of chondrocyte markers, mutant mice and littermates were sacrificed at 1 week of age, and tibiae were excised and freed of adherent tissue. Under a dissection microscope, GPs from the proximal tibiae were excised for RNA isolation and analysis. **Figure 7a** shows that TrkB mRNA was reduced to 15±1.9% in proximal tibial growth plates from TrkB mutant mice as compared to those from their floxed littermates, and the expression of p38α was reduced to 51±2.2% . The expression levels of the other p38 isoforms (ß, γ and δ) were unchanged. Col2a1 and ColX expression were reduced in the mutant mice to 55±3.6%, and 19±2.8% of wild type levels, respectively. Runx2 expression was reduced to 22±2.9% and Sox9 to 51±5.4% of wild type levels.

The MAP14 mutant mice were also examined for differences in gene expression patterns at the GP of the proximal tibiae. As shown in **Figure 7b**, the expression of Col2a1 was not significantly reduced in the mutant mice, but ColX expression was reduced to 13±2.7%. As was seen in the TrkB mutant mice, the MAPK14 mutants displayed reduced expression of Runx2 and Sox9 to 35±5.1% and 38±5.6%, respectively. The expression of p38α was reduced to 14±0.8% that of normal whole proximal tibial GP in the MAPK14 mutant mice; TrkB expression was not affected. The expression levels of p38 isoforms β, γ and δ were very low in both the normal and mutant mice (not shown), suggesting that p38α is the predominant p38 MAPK present at the growth plate in mice. The fact that global knockout mice for either p38β, γ or δ were normally grown supports this conclusion [14], [15].

**Figure 8:**
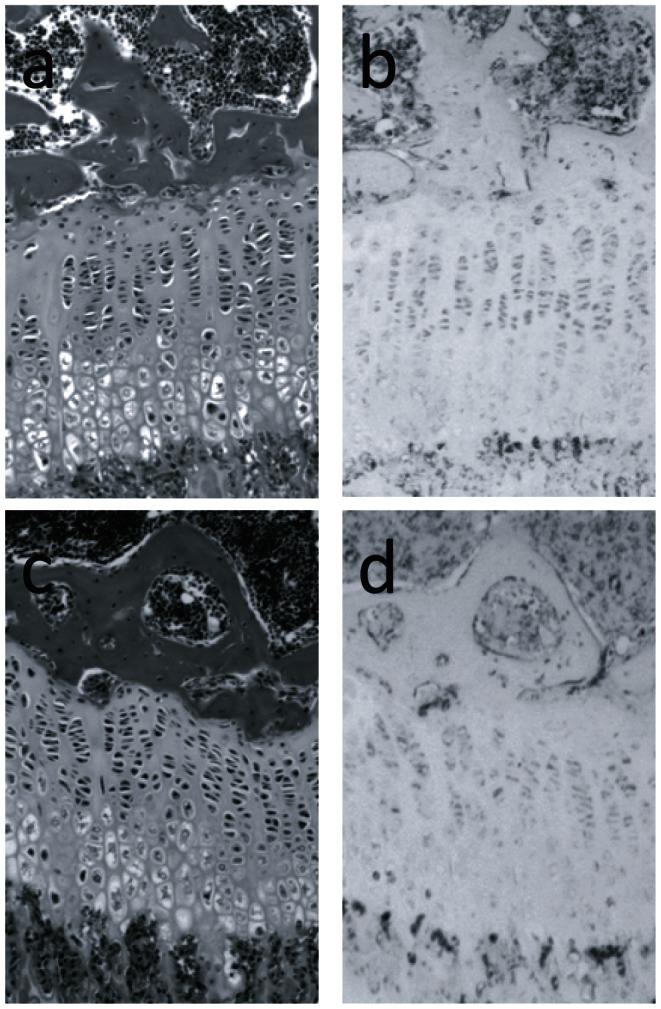
Histological analysis of TrkB mutants for phospho-p38. A, B, are stained histological sections from a 4 week old male *TrkB^loxp/loxp^* control; C, D are from a male *TrkB^loxp/loxp^;Col2a1-cre* littermate. A, C, H&E staining; B, D, phospho-p38 staining.

To further explore the connection between TrkB signaling and p38 MAPK activation, the proximal tibiae of 4 week old male *TrkB^flox/flox^* and *TrkB^flox/flox^;Col2a1-cre* mice were examined for differences in levels of active p38 protein. Immunohistochemistry performed with antisera against the dually-phosphorylated and active form of p38 showed that in normal mice p38 activity appears throughout the proliferative zone, with the greatest levels at the pre-hypertrophic zone (**Fig. 8**). The TrkB mutant mice, however, appear to have significantly reduced active p38 present at the GP, which is consistent with the reduced amount of total p38α mRNA seen in the GPs of TrkB mutant mice (**Fig. 7**).

### IGF-I levels are not affected in mutant mice

To rule out the possibility that the observed dwarfism in the mutant mice was due to perturbations in the growth hormone/IGF-I axis, serum from 8 week old mice was assessed for IGF-I concentrations by ELISA. The *TrkB^flox/flox^* mice had IGF-I levels that were not significantly different from that of the TrkB mutant mice, at 390±33 ng/ml and 375±26 ng/ml, respectively. Similarly, *MAPK14^flox/flox^* mice had serum IGF-I levels of 412±28 ng/ml, and the IGF-I levels in *MAPK14^flox/flox^ ;Col2a1-cre* mice were 403±31 ng/ml.

## Discussion

### The neurotrophin receptor TrkB is required for normal bone growth

BDNF was originally thought to be exclusively expressed in the CNS [19], where it stimulates neurite outgrowth and promotes neuronal cell differentiation and survival. BDNF is now recognized to be produced by a much wider variety of cell types, including vascular endothelial cells, leukocytes, adipocytes, and osteoblasts [8]–[10]. Studies showing that BDNF and TrkB are expressed in bone and cartilage, and that their expression is altered during bone healing, have implicated BDNF as a mediator of bone health [10], [20]–[23]. We have shown that TrkB is expressed in epiphyseal growth plate cartilage, and that BDNF positively regulates chondrocyte differentiation, suggesting that TrkB and BDNF also have a role in long bone growth.

In the present work we demonstrate the role of TrkB in longitudinal growth by removing TrkB (both full and truncated forms, see the Methods section) from the epiphyseal growth plates of mice. The mutant mice are dwarfed, with shortened tails and extremities due to a delay in hypertrophic differentiation within the growth plate. TrkB mutants have narrowed growth plates, largely due to narrowing of the hypertrophic zone, as proliferative capacity within less-differentiated cells is unaffected. These histological differences were apparent by 4 weeks of age in males. It is unclear why a significant number of the female mutant mice were severely dwarfed at birth. The *Col2a1-cre* transgene is active as early as 8.5 dpc [13], so it is possible that the severe dwarfism at birth is due to early disruption of TrkB expression in these mice. Some TrkB expression was detectible within the growth plates of all *TrkB^loxp/loxp^;Col2a1-cre* mice examined, whether by real time PCR analysis of RNA levels, or immunohistochemical methods. It is possible that the relatively mild dwarfism phenotype seen in most of the mice was due to retained expression of TrkB. Unfortunately we were unable to obtain histological samples from the severely dwarfed mice.

### TrkB is required for normal expression of transcription factors Sox9 and Runx2

That cell proliferation is preserved in the TrkB mutant mice is consistent with our previous observation in primary bovine chondrocytes, wherein inhibition of TrkB did not affect proliferation *in vitro* [4]. In the murine embryonal carcinoma-derived cell line ATDC5, TrkB inhibition with either K252a or AG879 dramatically blocks chondrocyte differentiation as measured by Col2a1 and ColX expression, but does not alter IGF-I-stimulated proliferation (4). The role of TrkB as a regulator of chondrocyte differentiation is apparent in the mutant mice that display reduced Col2a1 and ColX expression at the growth plate. We questioned whether the delayed hypertrophic differentiation in the TrkB mutant mice might involve altered expression of key transcriptional regulators, the transcription factors Sox9 and Runx2.

Sox9 is a master regulator of early cartilage development, and its inactivation in mouse embryos results in essentially no appendicular cartilage formation [24]. At the growth plate, Sox9 is expressed in reserve and proliferative zone cells, but is not present in more differentiated chondrocytes [25]–[27]. Heterozygous mutations in Sox9 cause a severe form of chondrodysplasia in humans called camptomelic dysplasia [28], [29]. The transcription factor Runx2 is required for osteoblast differentiation and early chondrogenic development [30], [31], and mice lacking Runx2 have almost no skeletal development [31]. At the growth plate, Runx2 is excluded from the proliferative zone, but re-activated at the pre-hypertrophic zone and throughout terminal hypertrophic differentiation of chondrocytes [32], [33]. In humans, haploinsufficiency of Runx2 causes cleidocranial dysplasia [34]–[36]. Runx2 is required for pre-hypertrophic and hypertrophic differentiation in mice [32], [37]. We found that in ATDC5 cells, TrkB inhibition greatly reduced the expression of both Runx2 and Sox9. Moreover, TrkB mutant mice have reduced expression of the transcription factors at the growth plate, with Runx2 at about 30% and Sox9 at 50% of normal, suggesting that TrkB might influence chondrocyte development at least partly by regulating the expression of these key transcription factors.

### Mice with a conditional deletion of p38α are similar to the TrkB mutant mice

We previously showed that BDNF-stimulated chondrocyte differentiation is dependent on p38 activity [4]. Of the four p38 isoforms, global deletions of either p38β, γ or δ result in mice with no discernible growth defect; however, p38α-deficient mice die at e10.5 due to defective placentation [14]–[16], [38], [39]. In an effort to demonstrate the role of p38α MAPK (MAPK14) downstream of TrkB in growth plate development, we deleted MAPK14 from the epiphyseal growth plates of mice, and found that the resulting dwarfism phenotype is very similar to that seen in the TrkB mutant mice. The p38α mutant mice also display dwarfism with shortened extremities and tails, and examination of tibial growth plate sections shows narrowing of both the proliferative and hypertrophic zones with preservation of chondrocyte proliferation. In cell culture, inhibition of p38 activity results in enhanced chondrocyte proliferation, and thus we anticipated that the MAPK14 mutant mice would have a widened proliferative zone and evidence of increased cell proliferation within that zone. However, the p38 mutant mice have growth plates with a reduced proliferative zone width. In our hands, the expression of p38α appears to be greatest in the pre-hypertrophic zone in normal mice, wherein cell proliferation ceases and differentiation begins. The expression of p38 at the pre-hypertrophic zone is consistent with the proposed role of p38 as a promoter of chondrocyte hypertrophic differentiation, rather than a regulator of proliferation *per se* [40]. To further cement the connection between TrkB and p38 activation, we also demonstrated that the level of activated p38 protein is decreased in the growth plates of TrkB mutant mice.

Chondrocyte-specific expression of constitutively active MEK6, the kinase just upstream of p38, also caused dwarfism in mice with a reduction of long bone length to about 80% that of control mice [41]. As seen in our p38α mutant mice, the transgenic mice expressing active MEK6 showed a delay in hypertrophic differentiation. Clearly p38 MAPK is involved in the regulation of hypertrophic differentiation, with either excessive or insufficient p38 activity being detrimental to the process.

### p38α is also required for normal expression of Sox9 and Runx2

Both Runx2 and Sox9 expression were reduced at the proximal tibial growth plate of p38α mutant mice to less than 40% that of their normal littermates. This result is consistent with the increase in Sox9 activity seen in the transgenic mice expressing active MEK6 [41]. We propose that the pro-differentiating activity of BDNF/TrkB operates through, at least partly, an increase in expression of Runx2 and Sox9 in response to p38 activation (Figure 8). Of note, both p38α expression and p38 phosphorylation is reduced in the growth plates of TrkB mutant mice, whereas TrkB expression is preserved in the p38α mutants. It is thus possible that the reduced expression of Runx2 and Sox9 in the TrkB mutant is mice is due to the reduced expression of p38α.

**Figure 9:**
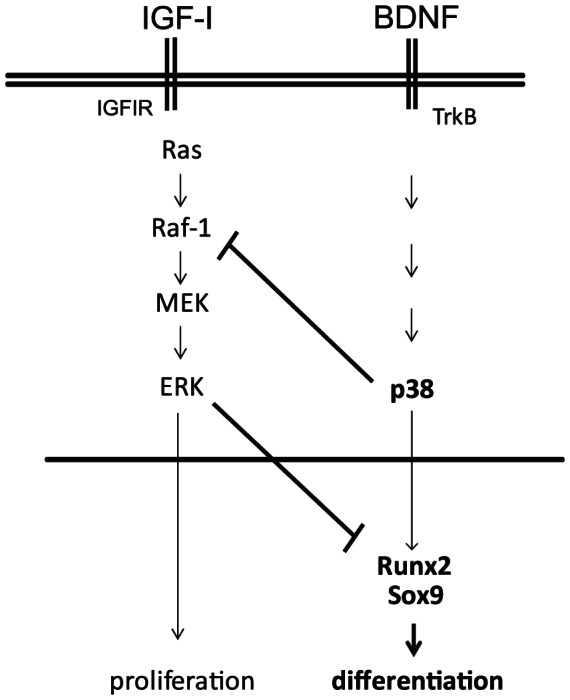
Proposed model of BDNF/TrkB regulation of chondrocyte differentiation via p38 activation. Unopposed IGF-I action favors suppression of Runx2 and Sox9 expression and proliferation, whereas BDNF binding to TrkB results in increased p38 activity, decreased ERK activity, increased Runx2 and Sox9 expression, and ultimately hypertrophic differentiation.

In summary, we propose a model wherein the neurotrophin receptor TrkB in growth plate chondrocytes is activated by BDNF to halt proliferation and promote differentiation via the activation of the MAPK p38. We have extended the model to include the key chondrocytic transcriptional regulators Sox9 and Runx2 as potential targets of the BDNF/TrkB/p38 pathway (**Fig. 9**). The current work validates the *in vitro* model, showing that TrkB and p38α are required for normal skeletal development and growth in mice. TrkB and p38α both play a role in chondrocyte differentiation, as proliferation was preserved, and hypertrophy was delayed at the growth plates of affected mice.

## Methods

### Generation of mice

*TrkB^flox/flox^* mutant mice [6] in a C57/B6 background (backcrosses to pure C57/B6 were performed initially) were crossed with mice carrying the *Col2a1-cre* transgene [13] also on a C57/B6 background to eliminate TrkB expression from cells of chondrocytic lineage. For the *TrkB* mutant mice, exon 1 of the TrkB gene, which encodes the signal peptide and the first 40 amino acids of the N terminus of TrkB, was floxed. In neural tissue, TrkB exists in its full-length form as well as truncated forms that lack the tyrosine kinase domain [42], [43]; the truncated forms of TrkB are capable of transmitting intracellular signals, and may have dominant inhibitory effects on BDNF signaling [44], [45]. In the *TrkB^flox/flox^* mutant mice, both full-length and truncated forms of TrkB are targeted.

To conditionally delete p38α from the growth plate, *MAPK14^flox/flox^* mice were obtained from the RIKEN Bioresource center (Tsukuba, Japan) and crossed with the *Col2a1-cre* mice.

In all crosses, only mice carrying a single *Col2a1-cre* transgene were used, as we noted that animals carrying two copies of the transgene appeared to have subtle differences in skull shape. All procedures involving animals were approved by the Institutional Animal Care and Use Committee at UT Southwestern Medical Center.

Genotype in each case was assessed by PCR of genomic DNA obtained from tail cuttings. Primers for cre were: 5′-GATGAGGTTCGCAAGAACCTG-3′ and 5′-ATCCGCCGCATAACCAGTG-3′; these primers produced a 300 bp product. Primers used for TrkB were: 5′-ACACACACAGTATATTTTACCA-3′ and 5′-CAAGAAGTCAGAGACCAGAGAGA-3′; approximately 300 bp and 500 bp products were produced for the WT and floxed gene, respectively. Primers for MAPK14 were: 5′-AGCCAGGGCTATACAGAGAAAAACCCTGTG-3′ and 5′-ATGAGATGCAGTACCCTTGGAGACCAGAAG-3′, producing 210 bp and 260 bp products, respectively

### Measurements

All mice were kept in a pathogen-free environment on a 12 h light-dark cycle. Litters were culled to a maximum of 7 pups per litter; once weaned, mice were housed at a maximum of 4 per cage. Water and standard chow were provided ad libidum. Starting at 7 days of age, weight, nose-to-tail length, and nose-to-rump length was measured twice weekly until 12 weeks of age; individuals performing measurements were blinded to genotype.

### Radiographic analysis

Male mice were sacrificed by isoflurane inhalation; skeletal morphology was imaged at 25 kVp for 0.4 sec using the small focal spot setting on a Lorad M III mammography system (Lorad Medical Systems, Danbury CT). Specific bones were measured using ImageJ [46].

### Histology

Tissues from 4 week-old mice were fixed in 4% paraformaldehyde/0.01 M PBS, pH 7.4 for 24 hours at 4°C. Tissues from mice 7 days of age or younger were decalcified for 1 week in 0.5 M EDTA, pH 8.0; specimens from older animals were decalcified for 2 weeks prior to imbedding in paraffin. 7 mm sections were cut and stained with hematoxylin and eosin (H&E). Immunohistochemical staining was done with either rabbit anti-rat collagen X at a 1∶50 dilution (Abcam, Cambridge, MA), or rabbit anti-human TrkB at 1∶100 (Abcam). Rabbit anti-p38 (Sigma) was used at 1∶100 dilution; rabbit anti-phospho-p38 (Cell Signaling) was used at 1∶50 dilution. Immunostaining was performed with the Cell and Tissue Staining Kit from R&D Systems (Minneapolis, MN) according to the manufacturer's instructions. For epiphyseal measurements (width, % width of hypertrophic zone), H&E stains analyzed by ImageJ by an individual blinded to genotype. Widths were analyzed at five points spaced evenly across the growth plate and the median values for width and %width of hypertrophic zone selected for each bone.

### BrdU uptake

Mice at 4 weeks of age were injected intraperitoneally with BrdU (100 µg/g body weight) and were sacrificed 4 hours later. Tibiae were fixed in 4% paraformaldehyde/0.01 M PBS, pH 7.4 for 24 hours at 4°C, decalcified in EDTA for 2 weeks, and embedded in paraffin. BrdU uptake in cells was determined by BrdU labeling with the BrdU Labeling and Detection Kit II from Roche Diagnostics (Indianapolis, IN). Cell proliferation was assessed as the percentage of positive cells in the proliferative zone within the microscopic field.

### ATDC5 cultures

ATDC5 cell culture and differentiation experiments were performed as described previously [17]. Briefly, cells were maintained in DMEM/F12 with 10% FBS (Invitrogen) plus penicillin/streptomycin/amphotericin B; once at confluence, differentiation was initiated by changing the medium to DMEM/F12 with 5% FBS plus 10 µg/ml insulin, 10 µg/ml human transferrin, and 10 ng/ml sodium selenite from Sigma (ITS). The medium was changed every other day. Protein kinase inhibitors were added between days 6–9. The inhibitors used were: p38 inhibitor SB203580 (Calbiochem, San Diego, CA) at 10 µM, TrkB inhibitors K-252a (Calbiochem) and AG879 (Calbiochem) at 3 nM and 10 µM, respectively. The PI3K inhibitor Wortmannin (Sigma) at 100 nM was also used as a general control, as we had previously shown that Wortmannin had minimal effects on ATDC5 cell development [47].

### RNA Isolation, cDNA Synthesis and real time RT PCR

ATDC5 cells were induced to differentiate with ITS; on day 6 post ITS addition, indicated kinase inhibitors were added. On day 9, cells were harvested into RNA STAT-60 (TelTest, Inc, Friendswood, TX), and RNA was extracted per the manufacturer's instructions. Tibial epiphyseal cartilage from 1 week old mice was dissected under a microscope and harvested into RNA STAT-60 as above. Genomic DNA was removed from each sample using DNA-Free (Ambion, Austin, TX), and 2 µg RNA was reverse transcribed with the High Capacity cDNA archive kit (Applied Biosystems). Real time RT PCR was performed using the Roche LightCycler 480 following the manufacturer's protocol. 18S detection was done using TaqMan in the LightCycler 480 Probes Master Mix (Roche) and 200 nM of each primer and probe. Other targets were analyzed using double-stranded DNA dye SYBR Green with the LightCycler 480 SYBR Green 1 Master Mix (Roche) and 200 nM of each primer. Primers spanned intron/exon boundaries wherever possible, and all RT-PCR reactions were confirmed to produce only a single PCR product by comparison of the melt curves at the completion of each PCR reaction. The primer sets for the mouse targets have been previously described [47]. Relative gene expression for each mRNA was calculated by the ΔΔCT method using the “control” sample (no ITS) as calibrator.

### IGF-I serum levels

Mutant and normal littermates of both genders were sacrificed at 8 weeks of age, and blood obtained via cardiac puncture. Serum IGF-I levels were assessed with the Mouse/Rat Quantikine ELISA kit from R&D Systems (Minneapolis, MN).

### Statistical Analyses

Data are expressed as means ±SD. For growth parameters, differences between *TrkB^flox/flox^* and *TrkB^flox/flox^;Col2a1-cre* mice and between *MAPK14^flox/flox^* and *MAPK14^flox/flox^*;*Col2a1-cre* mice was determined by one way repeated measures ANOVA, and was considered significant at *P*<0.05. For the RNA expression studies, significance between groups was determined by standard one-way ANOVA (SigmaPlot 11.0, SyStat Software, Inc. San Jose, CA). Differences were considered significant at *P*<0.05 unless otherwise noted.

## References

[1] Hunziker EB (1994) Mechanism of longitudinal bone growth and its regulation by growth plate chondrocytes. Microsc Res Tech 28: 505–519. 7949396 10.1002/jemt.1070280606

[2] Kronenberg HM (2003) Developmental regulation of the growth plate. Nature 423: 332–336. 12748651 10.1038/nature01657

[3] Nilsson A, Ohlsson C, Isaksson OG, Lindahl A, Isgaard J (1994) Hormonal regulation of longitudinal bone growth. Eur J Clin Nutr 48 (Suppl 1) S150–158; discussion S158–160. 8005082 10.1007/BF02558817

[4] Hutchison MR (2012) BDNF Alters ERK/p38 MAPK Activity Ratios to Promote Differentiation in Growth Plate Chondrocytes. Mol Endocrinol 10.1210/me.2012-1063 PMC3404299 22700586

[5] Klein R, Smeyne RJ, Wurst W, Long LK, Auerbach BA, et al (1993) Targeted disruption of the trkB neurotrophin receptor gene results in nervous system lesions and neonatal death. Cell 75: 113–122. 8402890

[6] Luikart BW, Nef S, Shipman T, Parada LF (2003) In vivo role of truncated trkb receptors during sensory ganglion neurogenesis. Neuroscience 117: 847–858. 12654337 10.1016/s0306-4522(02)00719-4

[7] Minichiello L, Calella AM, Medina DL, Bonhoeffer T, Klein R, et al (2002) Mechanism of TrkB-mediated hippocampal long-term potentiation. Neuron 36: 121–137. 12367511 10.1016/s0896-6273(02)00942-x

[8] Nakahashi T, Fujimura H, Altar CA, Li J, Kambayashi J, et al (2000) Vascular endothelial cells synthesize and secrete brain-derived neurotrophic factor. FEBS Lett 470: 113–117. 10734218 10.1016/s0014-5793(00)01302-8

[9] Yoshimura S, Ochi H, Isobe N, Matsushita T, Motomura K, et al (2010) Altered production of brain-derived neurotrophic factor by peripheral blood immune cells in multiple sclerosis. Mult Scler 16: 1178–1188. 20656764 10.1177/1352458510375706

[10] Yamashiro T, Fukunaga T, Yamashita K, Kobashi N, Takano-Yamamoto T (2001) Gene and protein expression of brain-derived neurotrophic factor and TrkB in bone and cartilage. Bone 28: 404–409. 11336921 10.1016/s8756-3282(01)00405-7

[11] Cobb MH (1999) MAP kinase pathways. Prog Biophys Mol Biol 71: 479–500. 10354710 10.1016/s0079-6107(98)00056-x

[12] Pearson G, Robinson F, Beers Gibson T, Xu BE, Karandikar M, et al (2001) Mitogen-activated protein (MAP) kinase pathways: regulation and physiological functions. Endocr Rev 22: 153–183. 11294822 10.1210/edrv.22.2.0428

[13] Ovchinnikov DA, Deng JM, Ogunrinu G, Behringer RR (2000) Col2a1-directed expression of Cre recombinase in differentiating chondrocytes in transgenic mice. Genesis 26: 145–146. 10686612

[14] Beardmore VA, Hinton HJ, Eftychi C, Apostolaki M, Armaka M, et al (2005) Generation and characterization of p38beta (MAPK11) gene-targeted mice. Mol Cell Biol 25: 10454–10464. 16287858 10.1128/MCB.25.23.10454-10464.2005 PMC1291241

[15] Sabio G, Arthur JS, Kuma Y, Peggie M, Carr J, et al (2005) p38gamma regulates the localisation of SAP97 in the cytoskeleton by modulating its interaction with GKAP. EMBO J 24: 1134–1145. 15729360 10.1038/sj.emboj.7600578 PMC556394

[16] Allen M, Svensson L, Roach M, Hambor J, McNeish J, et al (2000) Deficiency of the stress kinase p38alpha results in embryonic lethality: characterization of the kinase dependence of stress responses of enzyme-deficient embryonic stem cells. J Exp Med 191: 859–870. 10704466 10.1084/jem.191.5.859 PMC2195860

[17] Shukunami C, Shigeno C, Atsumi T, Ishizeki K, Suzuki F, et al (1996) Chondrogenic differentiation of clonal mouse embryonic cell line ATDC5 in vitro: differentiation-dependent gene expression of parathyroid hormone (PTH)/PTH-related peptide receptor. J Cell Biol 133: 457–468. 8609176 10.1083/jcb.133.2.457 PMC2120800

[18] Shukunami C, Ishizeki K, Atsumi T, Ohta Y, Suzuki F, et al (1997) Cellular hypertrophy and calcification of embryonal carcinoma-derived chondrogenic cell line ATDC5 in vitro. J Bone Miner Res 12: 1174–1188. 9258747 10.1359/jbmr.1997.12.8.1174

[19] Ernfors P, Bengzon J, Kokaia Z, Persson H, Lindvall O (1991) Increased levels of messenger RNAs for neurotrophic factors in the brain during kindling epileptogenesis. Neuron 7: 165–176. 1829904 10.1016/0896-6273(91)90084-d

[20] Asaumi K, Nakanishi T, Asahara H, Inoue H, Takigawa M (2000) Expression of neurotrophins and their receptors (TRK) during fracture healing. Bone 26: 625–633. 10831935 10.1016/s8756-3282(00)00281-7

[21] Pinski J, Weeraratna A, Uzgare AR, Arnold JT, Denmeade SR, et al (2002) Trk receptor inhibition induces apoptosis of proliferating but not quiescent human osteoblasts. Cancer Res 62: 986–989. 11861369

[22] Kurihara H, Shinohara H, Yoshino H, Takeda K, Shiba H (2003) Neurotrophins in cultured cells from periodontal tissues. J Periodontol 74: 76–84. 12593600 10.1902/jop.2003.74.1.76

[23] Kajiya M, Shiba H, Fujita T, Ouhara K, Takeda K, et al (2008) Brain-derived neurotrophic factor stimulates bone/cementum-related protein gene expression in cementoblasts. J Biol Chem 283: 16259–16267. 18390540 10.1074/jbc.M800668200 PMC3259653

[24] Akiyama H, Chaboissier MC, Martin JF, Schedl A, de Crombrugghe B (2002) The transcription factor Sox9 has essential roles in successive steps of the chondrocyte differentiation pathway and is required for expression of Sox5 and Sox6. Genes Dev 16: 2813–2828. 12414734 10.1101/gad.1017802 PMC187468

[25] Wright E, Hargrave MR, Christiansen J, Cooper L, Kun J, et al (1995) The Sry-related gene Sox9 is expressed during chondrogenesis in mouse embryos. Nat Genet 9: 15–20. 7704017 10.1038/ng0195-15

[26] Ng LJ, Wheatley S, Muscat GE, Conway-Campbell J, Bowles J, et al (1997) SOX9 binds DNA, activates transcription, and coexpresses with type II collagen during chondrogenesis in the mouse. Dev Biol 183: 108–121. 9119111 10.1006/dbio.1996.8487

[27] Zhao Q, Eberspaecher H, Lefebvre V, De Crombrugghe B (1997) Parallel expression of Sox9 and Col2a1 in cells undergoing chondrogenesis. Dev Dyn 209: 377–386. 9264261 10.1002/(SICI)1097-0177(199708)209:4<377::AID-AJA5>3.0.CO;2-F

[28] Wagner T, Wirth J, Meyer J, Zabel B, Held M, et al (1994) Autosomal sex reversal and campomelic dysplasia are caused by mutations in and around the SRY-related gene SOX9. Cell 79: 1111–1120. 8001137 10.1016/0092-8674(94)90041-8

[29] Foster JW, Dominguez-Steglich MA, Guioli S, Kwok C, Weller PA, et al (1994) Campomelic dysplasia and autosomal sex reversal caused by mutations in an SRY-related gene. Nature 372: 525–530. 7990924 10.1038/372525a0

[30] Ducy P, Zhang R, Geoffroy V, Ridall AL, Karsenty G (1997) Osf2/Cbfa1: a transcriptional activator of osteoblast differentiation. Cell 89: 747–754. 9182762 10.1016/s0092-8674(00)80257-3

[31] Komori T, Yagi H, Nomura S, Yamaguchi A, Sasaki K, et al (1997) Targeted disruption of Cbfa1 results in a complete lack of bone formation owing to maturational arrest of osteoblasts. Cell 89: 755–764. 9182763 10.1016/s0092-8674(00)80258-5

[32] Kim IS, Otto F, Zabel B, Mundlos S (1999) Regulation of chondrocyte differentiation by Cbfa1. Mech Dev 80: 159–170. 10072783 10.1016/s0925-4773(98)00210-x

[33] Yoshida CA, Yamamoto H, Fujita T, Furuichi T, Ito K, et al (2004) Runx2 and Runx3 are essential for chondrocyte maturation, and Runx2 regulates limb growth through induction of Indian hedgehog. Genes Dev 18: 952–963. 15107406 10.1101/gad.1174704 PMC395853

[34] Lee B, Thirunavukkarasu K, Zhou L, Pastore L, Baldini A, et al (1997) Missense mutations abolishing DNA binding of the osteoblast-specific transcription factor OSF2/CBFA1 in cleidocranial dysplasia. Nat Genet 16: 307–310. 9207800 10.1038/ng0797-307

[35] Mundlos S, Otto F, Mundlos C, Mulliken JB, Aylsworth AS, et al (1997) Mutations involving the transcription factor CBFA1 cause cleidocranial dysplasia. Cell 89: 773–779. 9182765 10.1016/s0092-8674(00)80260-3

[36] Otto F, Thornell AP, Crompton T, Denzel A, Gilmour KC, et al (1997) Cbfa1, a candidate gene for cleidocranial dysplasia syndrome, is essential for osteoblast differentiation and bone development. Cell 89: 765–771. 9182764 10.1016/s0092-8674(00)80259-7

[37] Inada M, Yasui T, Nomura S, Miyake S, Deguchi K, et al (1999) Maturational disturbance of chondrocytes in Cbfa1-deficient mice. Dev Dyn 214: 279–290. 10213384 10.1002/(SICI)1097-0177(199904)214:4<279::AID-AJA1>3.0.CO;2-W

[38] Tamura K, Sudo T, Senftleben U, Dadak AM, Johnson R, et al (2000) Requirement for p38alpha in erythropoietin expression: a role for stress kinases in erythropoiesis. Cell 102: 221–231. 10943842 10.1016/s0092-8674(00)00027-1

[39] Mudgett JS, Ding J, Guh-Siesel L, Chartrain NA, Yang L, et al (2000) Essential role for p38alpha mitogen-activated protein kinase in placental angiogenesis. Proc Natl Acad Sci U S A 97: 10454–10459. 10973481 10.1073/pnas.180316397 PMC27045

[40] Stanton LA, Sabari S, Sampaio AV, Underhill TM, Beier F (2004) p38 MAP kinase signalling is required for hypertrophic chondrocyte differentiation. Biochem J 378: 53–62. 14594450 10.1042/BJ20030874 PMC1223932

[41] Zhang R, Murakami S, Coustry F, Wang Y, de Crombrugghe B (2006) Constitutive activation of MKK6 in chondrocytes of transgenic mice inhibits proliferation and delays endochondral bone formation. Proc Natl Acad Sci U S A 103: 365–370. 16387856 10.1073/pnas.0507979103 PMC1326166

[42] Allen SJ, Dawbarn D, Eckford SD, Wilcock GK, Ashcroft M, et al (1994) Cloning of a non-catalytic form of human trkB and distribution of messenger RNA for trkB in human brain. Neuroscience 60: 825–834. 7936202 10.1016/0306-4522(94)90507-x

[43] Armanini MP, McMahon SB, Sutherland J, Shelton DL, Phillips HS (1995) Truncated and catalytic isoforms of trkB are co-expressed in neurons of rat and mouse CNS. Eur J Neurosci 7: 1403–1409. 7582115 10.1111/j.1460-9568.1995.tb01132.x

[44] Eide FF, Vining ER, Eide BL, Zang K, Wang XY, et al (1996) Naturally occurring truncated trkB receptors have dominant inhibitory effects on brain-derived neurotrophic factor signaling. J Neurosci 16: 3123–3129. 8627351 10.1523/JNEUROSCI.16-10-03123.1996 PMC2710135

[45] Baxter GT, Radeke MJ, Kuo RC, Makrides V, Hinkle B, et al (1997) Signal transduction mediated by the truncated trkB receptor isoforms, trkB.T1 and trkB.T2. J Neurosci 17: 2683–2690. 9092589 10.1523/JNEUROSCI.17-08-02683.1997 PMC6573096

[46] Schneider CA, Rasband WS, Eliceiri KW (2012) NIH Image to ImageJ: 25 years of image analysis. Nat Methods 9: 671–675. 22930834 10.1038/nmeth.2089 PMC5554542

[47] Hutchison MR, Bassett MH, White PC (2010) SCF, BDNF, and Gas6 are regulators of growth plate chondrocyte proliferation and differentiation. Mol Endocrinol 24: 193–203. 19897599 10.1210/me.2009-0228 PMC2802903

